# Current Overview on Clinical Management of Chronic Constipation

**DOI:** 10.3390/jcm10081738

**Published:** 2021-04-16

**Authors:** Jakub Włodarczyk, Anna Waśniewska, Jakub Fichna, Adam Dziki, Łukasz Dziki, Marcin Włodarczyk

**Affiliations:** 1 Department of Biochemistry, Medical University of Lodz, 92-215 Lodz, Poland; jakub.wlodarczyk@umed.lodz.pl (J.W.); jakub.fichna@umed.lodz.pl (J.F.); 2 Department of General and Colorectal Surgery, Medical University of Lodz, 90-549 Lodz, Poland; adam.dziki@umed.lodz.pl (A.D.); lukasz.dziki@umed.lodz.pl (Ł.D.); 3 Department of Normal and Clinical Anatomy, Medical University of Lodz, 90-752 Lodz, Poland; anna.wasniewska@umed.lodz.pl

**Keywords:** constipation, chronic constipation, pharmacology, surgery, clinical management

## Abstract

Constipation is one of the major gastrointestinal disorders diagnosed in clinical practice in Western countries. Almost 20% of population suffer from this disorder, which means constipation is a substantial utilization of healthcare. Pathophysiology of constipation is complex and multifactorial, where aspects like disturbance in colonic transit, genetic predisposition, lifestyle habits, psychological distress, and many others need to be taken into consideration. Diagnosis of constipation is troublesome and requires thorough accurate examination. A nonpharmacological approach, education of the patient about the importance of lifestyle changes like diet and sport activity state, are the first line of therapy. In case of ineffective treatment, pharmacological treatments such as laxatives, secretagogues, serotonergic agonists, and many other medications should be induced. If pharmacologic treatment fails, the definitive solution for constipation might be surgical approach. Commonness of this disorder, costs of medical care and decrease in quality life cause constipation is a serious issue for many specialists. The aim of this review is to present current knowledge of chronic constipation and management of this disorder.

## 1. Introduction

Constipation is one of the major gastrointestinal disorders diagnosed in clinical practice in Western countries. Worldwide prevalence is estimated from 12% to 19% [1,2,3]. Constipation is more frequent in North America and Europe in comparison with Asia, probably due to differences in culture, diet, or environment [2,4,5]. The definition of constipation points to a decreased number of defecations per week, as well as multiple other symptoms, such as sensation of incomplete evacuation, abdominal bloating, straining, elongated or failed attempts to defecate, hard stools, and necessity of digital disimpaction [3,6,7]. Constipation, due to its etiology, is commonly divided into two groups, primary and secondary. Primary constipation includes constipation predominant irritable bowel syndrome (IBS-C), functional constipation, slow transit constipation like myopathy, neuropathy, and functional defecation disorders. Secondary constipation may be a result of metabolic disorders (e.g., hypercalcemia), medications (e.g., calcium channel blockers or opiates), primary colonic disorders (e.g., cancer, proctitis) and neurologic disorders [3,5,8]. Constipation decreases life quality among patients, as they suffer from both physical symptoms and psychological distress. Many patients with constipation complain about dyspareunia, sexual dysfunction, and urine retention. Chronic constipation limits work productivity and social activities [4,5,9]. Diagnosing and treating constipation has a significant impact on economic burden, according to Sbahi et al. only testing for constipation costs almost $7 billion annually [4]. Commonness of this disorder, costs of medical care, and decrease in quality life cause constipation a serious issue for many specialists. The aim of this review is updating knowledge of chronic constipation and management in this disorder.

### Methods

The materials for this narrative review were searched for in the following databases: PubMed, Embase (OVIDversion), and Google Scholar. The search query consisted of the combination of the following keywords: “constipation”, “chronic constipation”, “irritable bowel syndrome”, “functional constipation”, “dyssynergic defecation”, “rectal evacuation disorders”, “outlet obstruction”, and “slow colon transit”. Results were limited to relevant papers published in English. There were no restrictions for the publication date for the articles cited in all subsections of the manuscript. The first search was performed on 22 October 2020, updated on 9 January 2021, with a final revision on 6 February 2021. References in all the included studies were reviewed for more eligible articles. Each article was reviewed independently by three (JW, AW, and MW) researchers for inclusion according to prior established inclusion and exclusion criteria. Disagreements on article selection were resolved through discussion until consensus was reached or resolved by discussion with JF and AD. Conference abstracts were excluded. Articles were excluded in the case of non-English language, inaccessibility of the full text, preclinical research, and commentaries. Extracted details included study population and demographics, details of interventions and controls, study methodology, and information to assess bias.

## 2. Pathophysiology of Chronic Constipation

The patients with chronic constipation may be divided into three groups according to the colonic transit [10]. Patients with normal colonic transit suffer from functional constipation, they are the most predominant subgroup. The second group consists of patients with rectal evacuation disorders, especially patients with dyssynergic defecation [11]. Dyssynergic defecation, also known as anismus, obstructive defecation, pelvic floor dyssynergia, or outlet obstruction, is a result of incoordination between the abdominal wall and pelvic floor muscles, and the anal sphincters [10,11]. This incoordination may be a result of several mechanisms that include paradoxical anal contraction, impaired rectal contraction and inadequate anal relaxation. The least common type of chronic constipation is slow colonic constipation. Patients from this group usually present with dysfunctional retrograde colonic propulsion or postprandial motor activity. Pathophysiology of constipation is multifactorial, thereby it is problematic to match exact factor to one of the above groups.

Positive family history of constipation and genetic predisposition seem to have a role in this disorder, since patients with functional constipation commonly have a positive family history, however there are no specific genes related to constipation.

Diet is one of the most relevant factors of constipation. Insufficient fiber or fluid intake results in constipation in all age groups [6,12]. Murray et al. reported that 19% of their patients who were suffering from chronic constipation, were also diagnosed with eating disorders, therefore it should always be taken into consideration during diagnosis [13,14]. Moreover, many reviews, especially focused on cow’s milk allergies in children, proved improvement in functional disorders after resignation from cow’s milk.

Another major factor of constipation is the lack of exercise. Inactivity may lead to obesity, what is yet another, commonly considered factor of constipation, nevertheless data about obesity and constipation are ambiguous [15,16].

Clinicians also consider a connection between dysbiosis of gut microbiota and constipation. IBS-C patients have a decreased number of *Actinobacteria* in fecal samples and increased level of *Bacteroides* in their mucosal samples, also treatment with the use of synbiotics, probiotics, prebiotics, antibiotics, and fecal microbiota transplantation improves condition of patients with constipation. Moreover, in contrary to patients with normal transit, patients with chronic constipation presented methanogenic bacteria producing more methane, which slows colonic transit. This is a compelling evidence of the impact of dysbiosis on constipation [17,18,19]. As a consequence of growing popularity of probiotics, some patients use *Bifidobacterium* or *Lactobacillus*. Nevertheless, in some trials, probiotics are still as efficient as placebo [5,10,20]. What is more, Yarullina et al. recently reported a lack of motor activity disturbance or dysbiosis in chronic constipation, challenging previous theories and compromising the efficacy of conventional probiotic treatment. However, their study was based only on 20 patients, which means that additional experiments of this matter are needed [21].

Efficient bowel movement depends on nerve endings of sensory neurons which react to the content of the intestine. Patients with constipation are frequently observed with colonic motor dysfunction, abnormal colonic sensation, and impaired defecation. The high-amplitude propagating contractions, that transfer content of the colon for the long distance, occur less frequently in patients with constipation [1,6,22,23]. Neuropathy and myopathy seem to be the most important causes of delayed colonic transfer. However, patients with neuropathy rarely receive satisfying treatment, whereas treatment of myopathic constipation is usually successful [5].

Studies prove connection between constipation and psychological factors: constipation is more common in children with attention deficit disorders and autism, and constipated patients are more often observed with anxiety, stress, trauma, and depression [6,24,25,26]. In addition, experience acquired while toilet training in infancy, or even earlier, has a strong impact on developing chronic constipation in the future. Some toddlers hide during defecating—this behavior may be a consequence of parental reactions to feces and its negative connotation in their culture. Due to that, children may feel ashamed or embarrassed, thus the usage of a toilet or potty is more complicated for them later. Children who were hiding during the defecation are in high risk of developing later toilet training and constipation [27].

Some patients with unpleasant associations of defecation, e.g., pain or social reasons try to avoid it through withholding the stool. That results in absorbing water by colonic mucosa from feces and leads to hard stools, what complicates the evacuation. Consequently, patients lose their regular urge to defecate [28]. Moreover, patients with normal colonic transit may develop symptoms of severe constipation, as a consequence of increased psychological distress [29].

Constipation is also associated with age and female sex [2,30]. Polypharmacy, inadequate fiber or fluid intake, life situations disturbing regular bowel movements, and decreased physical activity may also increase prevalence of chronic constipation in elderly people. Moreover, sexual hormones during pregnancy slow down peristalsis, and also females are at high risk of injury of the pelvic floor during labor [2,3,5,8].

Recently, more and more studies concerning chronic constipation focus on serotonin and its signaling role in the gut. Possible mechanisms involve a decrease in the number of Cajal cells or disruptions in the serotonin level. However, in the case of the latter it is still unclear whether the exact cause is in a decreased number of receptors and their function, or a decreased availability of serotonin at the receptors [31]. Nevertheless the serotonin signaling pathway is a target for multiple drugs used in the constipation treatment.

Other possible mechanisms of constipation involve influence of proto-oncogenes, overexpression of progesterone receptors, infectious agents, autoimmunity, and tyrosine kinase C [31].

## 3. Diagnosis

The first steps of diagnosis of constipation are: gathering detailed medical history and physical examination with particular attention to anal examination. This primary evaluation should simplify the identification of causes of constipation or confirm alarm symptoms if they are present [25,32] (Table 1).

The exact medical history should answer the questions about consistency, frequency, size of stools, sense of incomplete evacuation, abdominal bloating, straining, elongated or failed attempts to defecate, and the use of digital disimpaction. Changes in living conditions, medicaments, lifestyle changes, the duration, and the onset of symptoms are also relevant [3,5,6,7].

To diagnose functional constipation, presence of 2 from the Rome IV criteria (number a,b) with duration between 3 to 6 months is needed (Table 2). Rome IV criteria contains the Bristol stool form scale (BSFS) 1–2, what means that stool has a form of hard lumps/or nuts, and is difficult to defecate or stool is lumpy, sausage-shaped [29].

During abdominal examination, identifying abdominal masses or tenderness and presence of stool is possible [4]. In children, fecalith or hard stool are easily palpable, which simplify diagnosis of a fecal impaction, which occurs in most cases of functional constipation [33].

Digital rectal examination (DRE) is extremely significant part of the physical examination. During DRE pelvic floor dyssynergia is identified with 87% specificity and 75% sensitivity when standard manometry is used as a reference, whereas the specificity and sensitivity of rectal balloon expulsion are 56% and 80%, respectively [6,7,34,35]. Investigation of the anus may exclude alarm symptoms, indicating carcinogenesis process or other severe diseases of the gastrointestinal (GI) tract. Moreover, rectal examination reveals polyps, masses, hemorrhoids, anatomic abnormalities, excoriation, or halo symptom of the anus, which may be present in rectal prolapse. Rectal prolapse sometimes is not visible during examination in the left lateral position, then the patient should be moved to squatting position [34,36]. Moreover, the patient may feel a sharp pain that indicates an injury of mucosa, inflammation, ulcer, or fissure [32]. Clinicians should also pay attention to sphincter tone and incontinence [9]. If the condition of a constipated patient improves after usage of OTC laxatives or fiber supplements, no additional examination is recommended. On the other hand, colonoscopy is recommended for patients, who are at the age appropriate for colon-cancer screening, or with alarm signs [7,23,34]. However, due to increasing incidence of early-onset colorectal cancer, every clinician should consider this diagnosis also in constipated patients before they reach screening age [37].

### Laboratory Testing

If alarm symptoms are absent, the American College of Gastroenterology suggests not to perform routine blood test in chronic constipation. Clinicians may order chemistry panel, thyroid function tests or complete blood test to exclude or confirm metabolic diseases as a cause of constipation. Serologic tests for thyroid and coeliac disease are advisable in patients with unintentional weight loss, short stature, persistent gastrointestinal symptoms, or family history with positive first degree relatives history. Of note, in children with constipation testing for cow’s milk allergy, routinely is not recommended. Diagnosis should be individualized and based on symptoms [4,6,28,38].

**Figure 1:**
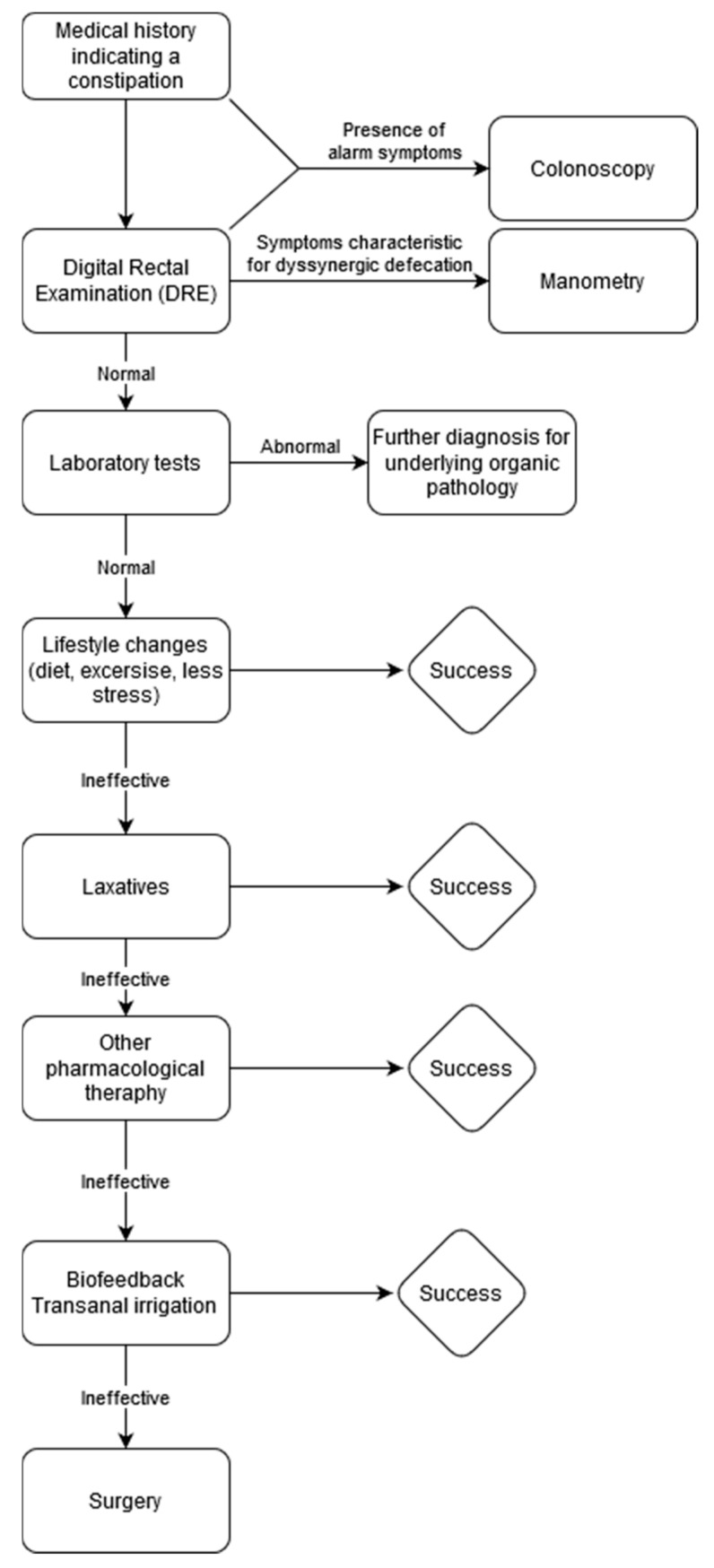
Management scheme of chronic constipation. In case of ineffectiveness of therapeutic approaches proposed strategies can be combined. Especially, Lifestyle Changes should be continued on every step.

To sum up and simplify the management of chronic constipation we propose scheme (Table 3 and Figure 1).

## 4. Management of Chronic Constipation

### 4.1. Nonpharmacologic Management

Nonpharmacologic treatment is the first-line management in constipation. This approach relies on educating patients about diet, fiber and fluid intake, physical activity, and toilet training.

Low intake of fiber leads to greater probability of constipation [41]. Dietary fibers are carbohydrate polymers and are digested in small extant in the colon. They may be soluble and insoluble, whereas soluble fibers are more effective [42,43]. One of the most often recommended is Psyllium. Studies confirmed its effectiveness in increasing defecation frequency, weight and consistency of stool, and lower tenderness of pain in defecation, however Psyllium does not improve colon transit [43]. Insoluble fiber, e.g., bran, improves intestinal transit time [42]. In the treatment of constipation, the recommendation is to gradually increase fiber intake, to a maximum of 35 g per day [43]. Unfortunately, fiber has side effects which result from gas production, such as: undesirable discomfort, flatus, or bloating [42]. Persistent gaseousness, which does not decrease after few days, needs switching one fiber supplement to another [7]. In addition, not all patients with constipation respond to treatment with fiber, the exceptions are patients with refractory constipation, slow transit constipation, or dyssynergic defecation [42].

Physical activity has positive influence on patients, moderate to vigorous activity alleviates symptoms and improves the quality of life in IBS [23]. Studies performed in Taiwan and Hong Kong proved a correlation between greater physical activity and increased bowel movements during one week [44,45], moreover studies from Iceland showed an association between higher physical activity and lower chance of constipation [46].

Treating constipation with increased fluid intake is only effective in dehydrated patients and poor water consumption is not associated with a higher risk of evacuation disorders [41,47].

Biofeedback therapy is especially effective in patients with defecatory disorders. The main purpose of this treatment is to restore normal pattern of defecation and to correct dyssynergia between abdominal, rectal, and anal sphincter muscles, and to increase rectal perception [48]. It uses instruments such as balloons, electromyography sensors, or manometry to train the patient how to use breathing to increase pressure in the abdomen, and how to coordinate relaxation of the anal and pelvic floor muscles during evacuation [32,49]. Biofeedback therapy might be done in an office or home setting, however home setting option is more cost-effective [43]. Many randomized clinical trials confirm an improvement in defecation and satisfaction of the patient [2]. Recent studies also suggest the use of electrical stimulation to induce neuromodulation of the colon and pelvic floor to permit defecation [2,50].

### 4.2. Pharmacological Management

#### 4.2.1. Laxatives

Laxatives, due to their effectiveness and availability, are the most commonly used pharmaceuticals in constipation. In case of failure of nonpharmacological management, laxatives are the first line of medications [51].

Osmotic laxatives change osmotic gradient in the intestine. As a result, secretion of water and electrolytes in the intestine is higher, ultimately volume of stool is increased and consistency is reduced [6,14,52]. They are usually well tolerated in long term use [32]. One of the commonly used osmotic laxatives is polyethylene glycol (PEG) [53]. PEG, in comparison with placebo, is significantly more effective in increasing the number of stools, lowering straining and alleviating the need for rescue laxatives. Studies comparing PEG and lactulose showed that PEG is more effective in treating constipation, while being better tolerated. Recommended dose of PEG per day is 10–20 g [34,54]. Despite great effectivity of PEG, it has adverse events, such as diarrhea or distension [29]. Lactulose, as well as PEG, is an osmotic laxative. Lactulose is not digested in the small intestine but is fermented by colonic bacteria, therefore it might induce bloating [34,43].

Saline laxatives, like magnesium citrate and sulfate (Ebson salt), disodium phosphate, sodium phosphate which transport water to the lumen of small and large intestine, also belong to the group of osmotic laxatives. Noteworthy, Dupont and Hebert suggest to drink magnesium sulfate-rich natural mineral water, due to its effectiveness, low costs and naturality, with the minimum daily intake of magnesium sulfate at 20 mmol [52]. Nonetheless, consumption of mineral water and other saline laxatives should be carefully considered in elderly patients or at risk of cardiovascular diseases, renal impairment, and hypertension [29,52].

Another group are stimulant laxatives that directly stimulate intestinal motility by Auerbach and myenteric plexus, and increase water and electrolyte secretion to the intestinal lumen [52,53]. Stimulant laxatives are usually prescribed as the next step in the management of functional constipation [43]. These laxatives are mostly used as a rescue therapy, when there are no bowel movements for 3 days [3]. Stimulant medications should be taken about half an hour after breakfast for the purpose of synchronizing gastrocolic response with medication effect [23]. The common examples of this group are sodium picosulfate, senna and cascara (both present in teas), and the most popular bisacodyl [53]. A randomized clinical trial confirmed the superiority of bisacodyl to sodium picosulphate and placebo [55]. Evidence of the effectiveness of solely senna is questionable, but connection of fiber and senna improves constipation in comparison with placebo [56]. Stimulant laxatives in persistent use do not seem to cause rebound constipation or tolerance on termination of treatment or injury to the colon [34]. The most frequent adverse effects are cramping and diarrhea [29]. The prevalence of side effects of bisacodyl declines over time. After first week of therapy side effects occur in 57% patients, whereas, after the fourth week, only in 5% [43].

Docusate, lubricants (liquid paraffin, prokinetic laxative), and emollients are other types of laxatives [5]. Mineral oil is the most often prescribed lubricant laxative, which softens stool and lowers water absorption [32].

The common belief is that permanent use of laxatives may cause tolerance and habituation. However, studies show that these conditions reveal among patients with slow colonic transit in whom other types of laxative agents are inefficient [51].

Even though there is a wide range of laxatives, almost 50% of patients who take these medicines are not satisfied with the results [9]. Therefore, it is crucial to develop new agents based on more physiological mechanisms in order to treat a larger share of constipated subjects.

#### 4.2.2. Secretagogues

Prosecretory agents treat constipation by modulating ion channels of epithelium to enhance colonic secretion and improve colonic transit [43]. Intracellular synthesis of cGMP is stimulated by activation of the guanylate cyclase C receptor, later the cGMP-dependent protein kinase II is promoted to phosphorylate cystic fibrosis transmembrane conductance regulator, thereby growing secretion of chloride and paracellular movement of water and sodium into the intestinal lumen [57].

Lubiprostone opens the chloride channel protein 2 and causes secretion of the chloride into intestine. Randomized controlled trials showed that lubiprostone (24 µg twice per day) increases spontaneous bowel movements within 24 to 48 h after first dose. The most frequent adverse effects of lubiprostone, which depend on dose, are nausea, diarrhea, and headache [43,57].

Linaclotide binds and activates guanylate cyclase-C receptors what leads to sodium absorption and chloride and bicarbonate secretion [58]. Linaclotide is successful in achieving spontaneous bowel movements and complete spontaneous bowel movements in constipated patients and patients with IBS-C [59]. The most common side effect of linaclotide is diarrhea which may occur during the first 4 weeks of therapy [60].

Plecanatide may also activate guanylate cyclase-C receptors, thus it has similar effects as linaclotide. Plecanatide, 2 mg once daily, effectively improves the quality of life in patients due to increased spontaneous bowel movements [58]. Diarrhea is a rare side effect but it may lead to discontinuation of therapy [61].

Tenapanor is an inhibitor of the sodium and hydrogen exchanger isoform 3 [43]. Tenapanor, by decreasing the absorption of phosphate and sodium, increases intestinal transit, fluid, and the number of the defecations, it also relieves abdominal pain. The most common side effect of tenapanor is diarrhea, whereas abdominal distension, dizziness, rectal bleeding and flatulence are less frequent [62]. The recommended dose of Tenapanor is 50 mg twice daily.

#### 4.2.3. Serotoninergic Agonists

Serotonin agonists, by activating 5-hydroxytryptamine receptor 4 (5-HT4) located in the gastrointestinal tract, stimulate secretion of the intestine and its motility [32].

Prucalopride is a highly selective agonist of the 5-HT4 receptor, it is well tolerated and safe for patients with cardiovascular diseases. In a paper from 2016, prucalopride was successful in increasing the number of bowel movements per week [63]. Moreover, prucalopride improves anorectal and abdominal symptoms such as pain, bloating, and distension [9]. Daily dose of prucalopride is 2 mg. Side effects are rare: headache, abdominal pain, nausea, and diarrhea were observed [64].

Velusetrag is a new agent, which has high intrinsic activity and a high degree of selectivity for the 5-HT4 receptor [38,64]. By accelerating the gastric emptying and reducing the intestinal and colonic transit time, velusetrag is considered as an effective treatment for gastrointestinal motility disorders, including constipation [64]. Velusetrag, in current studies, does not present interactions with other medications and does not lead to adverse effect on cardiovascular system, nevertheless further research should be conducted.

Some of the 5-HT4 agonists (tegaserod, cisapride) are currently restricted in constipation due to possible harmful side effects on cardiovascular system [32].

#### 4.2.4. Probiotics and Prebiotics

To this point, various probiotic species and strains have been suggested as beneficial agents regarding gastrointestinal diseases and gut motility. Many studies confirm their effectiveness in adult patients with constipation, based on improvement in intestinal transit and stool consistency, increased evacuation frequency, and decreased flatulence [65]. Moreover, studies showed that the effects of probiotics are species- and strain-specific, with indication that multispecies strains with *Bifidobacterium lactis* have the most promising beneficial effects [66]. However, due to large heterogeneity and high levels of bias among studies and lack of randomized controlled trials it is burdensome to provide specific guidelines. Current evidence does not suggest precise strain, which is the most clinically efficacious. Moreover, a recent paper by Harris et al. featured that conclusions from studies regarding probiotic treatment of functional constipation in children resulted from methodological errors, highlighting the susceptibility of evidence synthesis to oversights in study selection, quality assessments, and data extraction and collation [67].

Although there is no clear consensus on probiotics as a constipation agent, a recent survey of 1830 health professionals revealed that 18% recommend probiotics to patients with constipation [68].

#### 4.2.5. Other Agents

Patients regularly treated with opioids are challenging group in terms of chronic constipation treatment. Estimated percentage of patients who suffer from constipation after the use of opioids, which slow down the intestinal transit, ranges from 50% to 90%. For the treatment of the opioid induced constipation and ileus, peripherally acting mu-opioid receptor antagonists are used. They do not disturb analgesic effect, but only improve opioids adverse effect linked with GI tract, such as constipation, nausea, and vomiting [69]. This group is represented by Methylnaltrexone, Alvimopan, and Naloxegol. Methylnaltrexone bromide is dedicated for patients who are receiving palliative care with opioid induced constipation and advanced illness, after laxative therapy, which was insufficient. Recently, it was also approved for opioid induced therapy in adult patients with chronic non-cancer pain [4,70].

Elobixibat increases bile acid concentration in the gut, through the suppression of bile acid transporters, which leads to improvement of intestinal transit time and occurrence of defecation [71]. Recent retrospective study showed 74% improvement rate in the spontaneous bowel movement [72]. Elobixibat is generally safe medication, however it may cause mild disorders such as diarrhea, distension, abnormalities in liver function tests, abdominal pain, or nausea [73]. Chenodeoxycholic acid was also originally prescribed for treating gall stones and later used as a drug for treating constipation due to its adverse effect [6].

Colchicine is mainly used in arthritis but due to its side effect, which is diarrhea, may be beneficial in treating constipation [74,75]. Colchicine reduces intestinal transit time and increase spontaneous bowel movements. Long-term therapy of colchicine has minor side events (diarrhea, nausea), whereas neuropathy and myopathy occur very rarely [74].

Misoprostol is an analog of prostaglandin E2, which is approved as a prevention of gastric ulcers induced by anti-inflammatory drugs. Misoprostol improves frequency of defecation, increases intestinal transit time and the weight of stools. On the other hand Misoprostol may induce abdominal bloating, cramping, or nausea. Among women at reproductive age may cause spontaneous abortion, birth defects, or premature birth, thus the U.S. FDA (Food and Drug Administration) did not accept it as a treatment for constipation [57].

Another treatment option for constipation was recently presented by Fabisiak et al. According to their meta-analysis, cannabinoid 1 receptor (CB1) inverse agonists cause diarrhea and may be candidates for the treatment of constipation-predominant IBS and chronic constipation. Nonetheless prospective clinical trials on the possible targeting of CB1 and the endocannabinoid system are warranted [76].

Each patient requires an individually planned treatment approach, therefore it is difficult to provide universal treatment algorithm or specific guidelines. Absence of standardized and validated drugs comparisons makes it difficult to define fixed recommendations. However, there are other relative efficacy measures for mentioned agents (Table 4). Number needed to treat is a useful indicator, although it is limited by discrepancies in patient populations, end points, and duration of treatments between studies.

### 4.3. Surgical Approach

When pharmacological treatment fails, the implementation of surgical interventions should be considered. Whereas approach is strongly diversified, it mostly depends on physicians, who try to apply the least radical solution [6]. Patients with slow-transit constipation may be treated with ileostomy, total colectomy with ileorectal anastomosis (after removal of the colon, the ileum is connected to rectum), cecostomy with antegrade enemas. Patients with outlet rectal obstruction syndrome mostly require repair of rectoceles or surgery for rectal intussusception. Those with combination of dyssynergic defecation and slow-transit constipation may benefit from a segmental colonic resection or total colectomy, but pelvic floor dysfunction should be corrected first. Ileostomy is a surgical intervention that may relieve uncorrectable, severe pelvic floor dysfunctions. However, segmental colectomy, instead of total colectomy is a short-term solution and constipation may relapse.

Antegrade continence enemas may be used to achieve anterograde irrigation of the colon. Antegrade continence enemas enable rinsing of the fluids with or without laxative agents through an external-opening aperture toward the colonic lumen. The most frequent procedures to establish this opening are the Malone procedure (through an appendiceal conduit) and percutaneous cecostomy (PEC). PEC is preferred over colectomy in patients with significant comorbidities who have higher surgical risk. PEC may be performed under conscious therapy or local anesthesia, whereas 30% of patients after Malone appendicectomy have complications [83].

Surgery usually is shown to be associated with a higher degree of patient satisfaction, but nearly half of them complain about abdominal pain after surgery [83]. Proper surgical procedure implementation requires individualized approach and high expertise of surgeon in selected technique.

#### 4.3.1. Colonic Resection

Recently, metanalysis regarding outcomes and morbidity of colonic resections as a treatment approach for severe and persistent constipation provided valuable data [84]. Complications occurred in 24% of patients, while the mortality rate was 0.4%. Recurrent episodes of small bowel obstruction occurred in about 15% of patients in the long-term, which lead to necessary reoperation. Most patients reported a satisfactory or good outcome after colectomy, whereas in a minority of patients the negative long-term functional outcomes may persist. Recently it is stated that colectomy for chronic constipation may benefit some patients, but at the cost of substantial short- and long-term morbidity. Possible complications include diarrhea, incontinence, and bowel obstruction. According to a Rotholtz and Wexner results of segmental colonic resections for constipation are disappointing compared to ileorectal anastomosis [85].

#### 4.3.2. Surgery for Outlet Obstruction Syndrome

Obstructed defecation syndrome (ODS) is a condition that is frequently caused by rectocele or rectal intussusception. According to Hicks et al. more than 90% of patients who underwent surgery due to rectocele or rectal intussusception had significant improvement of symptoms, regardless of which type of surgery was performed [86]. However, surgical indications for ODS are poorly defined, and surgery should be considered only if functional significance can be determined [87]. Currently there is broad variety of surgical techniques including Delorme operation, ventral mesh rectopexy with or without resection, and stapled transanal rectal resection (STARR). However, still based on current data, it is unclear which approach provides best results with low risk of side effects.

Despite properly carried selection of cases for surgery, postoperative early and late morbidity can still be expected. Eventually, before surgery, a generalized gastrointestinal motility disorder should be excluded [49].

#### 4.3.3. Sacral Nerve Stimulation in Obstructed Defecation Syndrome and Slow Transit Constipation

Recent studies including a Cochrane meta-analysis implies that sacral nerve stimulation is still considered not to be an effective solution [34]. Results from studies noted high morbidity rates ranged between 13% and 34%, with overall device removal rate between 8% and 23%, while the success rate of this treatment was typically 57–87% for patients receiving permanent implants. Moreover, according to another study, which noted that the 5-year success rate for isolated constipation was only 31.2%, whereas another study reported reduction in VAS (visual analog scale) scores after 5 years follow-up on a 37.1% level [88,89].

### 4.4. Other Possibilities of Treatment

#### 4.4.1. Acupuncture

Acupuncture and electro-acupuncture is Chinese treatment modality [90]. Patients with severe constipation may be treated with acupuncture, however they should not have any comorbidities, and their constipation should not be caused by medications [90,91].

#### 4.4.2. Fecal Microbiota Transplantation (FMT)

Owing to dysbiosis of gut microbiota being a possible cause of constipation, FMT was suggested to be a method of treatment. According to Ding et al. this therapy seems to be efficient, but of short-duration; the effect of treatment was present up to 24 weeks [92]. Another recent study showed that clinical cure rate of FMT was 73.5%, whereas clinical remission rate (assed after 3 month follow-up) was 14.7% [93]. Nonetheless, according to Ohara FMT may successfully colonize the recipient gut by donor microbiota up to 1 year after transplantation [94].

#### 4.4.3. Massage

An alternative, but also effective nonpharmacological method, is abdominal massage. According to Sinclair, abdominal massage may accelerate colonic transit time, stimulate peristalsis and lower the pain and discomfort that often occur with constipation [1]. This management might be successful for patients with slow transit constipation, chronic and long-term functional constipation. Advantages of massage are a lack of side-effects, high efficiency, and low costs. Patients undergoing this treatment need fewer pharmacological agents [95,96]. On the other hand, manual massage is time-consuming and requires care, what may be cumbersome. One of the latest alternative method is an automatic abdominal massage device [95].

## 5. Conclusions

Constipation is a common and complicated disorder, where accompanied symptoms are not only distressing but also decrease the quality of life. Pathophysiology of constipation is complex and multifactorial. Diagnosis is based on predefined symptoms and Rome criteria, therefore, physicians should especially concentrate on a medical interview. After data from interview are collected, clinicians have several possibilities of diagnosing constipation with the use of the latest technologies. First-line therapy is a nonpharmacological approach. The main role of the physician is to educate the patient about the importance of the diet, fiber intake, and physical activity. In case of ineffective treatment, pharmacological approach should be induced. There is a wide range of pharmacological agents such as laxatives, secretagogues, serotonergic agonists, and many other medications, however every drug has its advantages and disadvantages that should be considered before prescription. If pharmacologic treatment fails, the definitive solution for constipation might be surgical approach. Commonness of constipation, costs of medical care, and a decrease in the quality of life cause this disorder a serious nuisance for many specialists, thus further attention must be given to development of medications to increase effectiveness, decrease their side effects, and the probability of drug-drug interactions.

## Figures and Tables

**Table 1:** Alarm symptoms to be excluded during diagnosis of chronic constipation [6,9].

Alarm Symptoms
Unintentional weight loss (more than 10% during 3 months)
Stools with blood Positive family history of inflammatory bowel disease or colonic cancer
Rectal tenesmus
Iron deficiency anemia
Jaundice
New-onset symptoms after 50 years of age
Positive fecal occult blood test
Cachexia

**Table 2:** Rome IV criteria for functional constipation [29].

Rome IV Criteria
Must include 2 or more of the following fulfilled for the last 3 months with symptom onset at least 6 months prior to diagnosis Straining during more than one-fourth (25%) of defecations Lumpy or hard stools (Bristol stool from scale 1–2) during more than one-fourth (25%) of defecations Sensation of incomplete evacuation during more than one-fourth (25%) of defecations Sensation of anorectal obstruction/blockage during more than one-fourth (25%) of defecations Manual maneuvers to facilitate more than one fourth (25%) of defecations (eg, digital evacuation, support of the pelvic floor) Loose stools are rarely present without the use of laxatives
Insufficient criteria for irritable bowel syndrome

**Table 3:** Possible advanced diagnostic approaches.

Diagnostic Tool	Description	Diagnosis	Comments
Magnetic Resonance (MR), Computed Tomography (CT), Endoscopy	MR and CT are imaging studies, while colonoscopy is endoscopic examination. All three examinations are used to identify different pathologies in gastrointestinal tract.	Characterization of the specific etiologies and complications of constipation is facilitated by both anatomic and functional imaging which range from basic radiography to MR imaging and endoscopy.	Procedures including colonoscopy, MR and CT can be very effective in obtaining evidence for the cause of unexplained symptoms, the use of chronic laxative and possible mucosal lesions. However, patients during CT are exposed to ionizing radiation.
Balloon Expulsion Test (BET)	Measures time required for a patient in a seated position to evacuate a balloon filled with water or air; instead of balloons artificial stool may be used [39,40].	Normal expulsion time varies from 1 min to 5 min, however sensitivity and specificity is higher for 2 min cut-off in constipation. Longer time is characteristic for patients suffering from dyssynergic defecation.	Even though, the test is highly sensitive and specific for defecatory disorders, the results might be falsely negative in some patients, for example with pelvic laxity (rectocele, sigmoidocele etc.) [23,39]. Other patients may strain overly and eject the balloon and the result does not express normal functions of their anus and rectum.
Anorectal manometry (AM)	Assesses anal and rectal pressure during attempted defecation and at rest. AM also measures rectal sensation, compliance and rectoanal reflexes [41].	2 out of 5 abnormalities should be present during manometry to recognize defecation disorders: Anal pressure during defecation > 90th percentile Anal pressure in rest > 90th percentile Anal relaxation < 10th percentile Rectal pressure < 10th percentile Rectoanal gradient < 10th percentile [23]	AM is mostly suggested to diagnose dyssynergic defecation, it is especially dedicated to confirm Hirschsprung disease. When a patient attempts to defecate normally, pressure in rectum rises. This increase is synchronized with a decrease in anal sphincter pressure, mainly because of relaxation of the external anal sphincter. This maneuver is voluntary. The inability to achieve this coordination is mostly observed in patients with dyssynergic defecation. Conventional AM collects data from single points, whereas high-resolution AM collects circumferential data, from the whole anal canal and distal part of the rectum. To reduce artificial movements and to improve spatial resolution of anorectal pressures, high-resolution AM catheters are used [42]
Rectal Sensation Testing (RST)	Simple test with the use of balloon that is attached to a catheter, and the balloon in the rectum is filled with air. Patient reports first sensations, desire to evacuate, urgency and maximum of their tolerance [34].	Reduced sensitivity of the rectum to distention indicates hyposensitivity (e.g., in constipation predominant subtype of irritable bowel syndrome (IBS-C) or spinal cords injuries), whereas increased sensation indicates hypersensitivity (e.g., fecal urgency, diarrhea predominant IBS, ulcerative colitis).	Results may be affected by biomechanic or structural properties of the rectum.
Defecography	Contrast material (150 mL) in rectum allows to investigate anorectal region during defecation and at rest [3,4].	Usually performed in case of a discrepancy between clinical impression and AM, or when structural abnormalities are assumed [7]. The test may reveal long time of retention of contrast material, decreased activation of levator muscles, absence of a stripping wave in the rectum or inability in expulsion of the barium.	The drawback of this examination is radiation exposure, patient embarrassment, inconsistent methodology, and limited availability. Due to these disadvantages defecography is not performed routinely [3,4].
Colonic Transit Study (CTS)	There are 3 methods of assessment stool transit: Colonic transit scintigraphy. Abdominal radiography of the ingestion of radiopaque marker. Tracking of the movement of the ingested wireless motility capsule [4].	Scintigraphy: Colonic transit is evaluated on the basis of the geometric center (GC). GC is the weighted average of the isotope distribution within the colon and stool. Patients with GC at 24 h less than 1.7, and GC at 48 h less than 3.0 are considered to have slow transit. Radiography: Decreased transit time is defined as presence on an abdominal X-ray of more than 5 markers in the colon after 5 days after capsule ingestion [3]. Wireless motility capsule: Slow transit is diagnosed above the 95th percentile of the normal subjects, what is identified as 59 h [43].	By reason of high costs, safety and availability, abdominal radiography of the ingestion of radiopaque marker is the preferred method. A CTS cannot differentiate between patients with slow-transit constipation and isolated dyssynergic defecation [3].
Fecobionics	An artificial stool which collects dynamic measurements of multiple variables in the process of defecation in a single examination. This technology provides integrated measurements of bending angle, pressure and geometric profiles with assessment of sensation [44].	Possible application in diagnosis of chronic constipation and fecal incontinence and in dyssynergic defecation in biofeedback training [45].	Fecobionics is still a research technology, however it has a vast potential.
Neurophysiology testing and electromyography	Records the activity of the muscle of the anal sphincter [34].	Diagnosing neuronal innervation and providing information about neuromuscular function. Abnormal activity may be an evidence for denervation, which is present in injury of the pudendal nerve or cauda equina syndrome.	Both tests are rarely used, because of limited availability. Moreover, they are troublesome and painful examination, thus it is performed only in a few research centres.

**Table 4:** Comparison of pharmacological agents used in the treatment of chronic constipation.

Drug	Dose	Number Needed to Treat
PEG [77]	17 g, daily	2 (95% CI: 1–3)
Lactulose [78]	20 g, daily	4 (95% CI: n/a)
Bisacodyl [79]	10 mg, daily	4 (95% CI: n/a)
Lubiprostone [80]	24 μg, twice daily	4 (95% CI: 3–6)
Linaclotide [20]	145 μg, daily	5.6 (95% CI: n/a)
Prucalopride [77]	2 mg, daily	6 (95% CI: 5–9)
Velusetrag [81]	15 mg, daily	3.4 (95% CI: n/a)
Elobixibat [82]	10 mg, daily	3 (95% CI: n/a)

PEG, polyethylene glycol; CI, confidence interval.

## Data Availability

No new data were created or analyzed in this study. Data sharing is not applicable to this article.
